# Theoretical Analysis and Design of Magnetostrictive Lamb Wave Detection

**DOI:** 10.3390/s26123824

**Published:** 2026-06-16

**Authors:** Jing Zhang, Wei Liu, Minghui Bao, Chao Yang, Jiahao Dai, Zhihong Fu

**Affiliations:** 1 State Key Laboratory of Power Transmission Equipment and System Security and New Technology, School of Electrical Engineering, Chongqing University, Chongqing 400044, China; zhangjbbs123@163.com (J.Z.);; 2 Professional Technology Center, Chongqing Electric Power Company Electric Power Science Research Institute, Chongqing 401123, China

**Keywords:** magnetostriction, mechanism model of excitation source, Lamb wave detection, defect detection in flat steel of down conductors

## Abstract

Conventional piezoelectric transducers suffer from stringent coupling demands and poor environmental robustness, limiting their utility for defect detection in the flat steel of down conductors in grounding grids. To overcome this, this study presents a Lamb wave excitation source based on magnetostriction. A mechanism model of the excitation source is established by analyzing the coupling among coil-driven electromagnetic excitation, magnetostrictive deformation, mechanical loading, and Lamb wave propagation in flat steel. The excitation source configuration and magnetization scheme are designed according to the geometric features of the down conductor. The experimental results show that, under pulsed current excitation, the magnetostrictive material produces a transient mechanical response, injecting disturbances into the flat steel and thereby enabling Lamb wave detection. The proposed source is compact and robust, showing strong potential for field applications. This study provides a novel active guided-wave solution for defect detection in the flat steel of down conductors and lays a foundation for subsequent signal analysis and engineering practice.

## 1. Introduction

As a kind of elastic guided wave that propagates in thin plates with free boundaries, Lamb waves are widely used in defect detection and health monitoring for metal plate structures due to their characteristics of long-distance propagation and high sensitivity. Numerous studies have shown that Lamb waves are formed by the multiple reflections and coupling of longitudinal and shear waves at the upper and lower surfaces of the plate, exhibiting two typical vibration modes: symmetric (S mode) and antisymmetric (A mode) [1]. Among these, due to its large out-of-plane displacement and strong coupling effect with common thickness-direction defects, the A0 mode is the most sensitive and valuable mode for low-frequency Lamb wave detection [2]. However, the dispersion effect causes different frequency components to propagate at different velocities, broadening the signal in the time domain and making defect identification difficult. Furthermore, energy attenuation during Lamb wave propagation results from multiple factors, and it directly affects the detection range and propagation distance. These factors include intrinsic material attenuation, where the viscoelasticity or internal friction of the material itself converts energy into heat, which is particularly significant in polymers and composite materials; scattering attenuation caused by the microstructure also leads to energy diffusion [3]. Geometric factors also contribute to attenuation, primarily including energy leakage when the structure contacts an external medium [4], and mode conversion and energy redistribution caused by structural discontinuities such as curvature and welds [5,6]. Attenuation directly determines the rate at which the signal-to-noise ratio decreases with distance. Excessive attenuation can cause noise to overwhelm the effective signal, thereby severely limiting the detection range [7].

Regarding excitation methods, most studies employ the oblique incidence method or the normal coupling method to generate Lamb waves. Among these, normal coupling can effectively produce a superimposed wave field of multiple low-frequency modes and is suitable for applications involving direct surface excitation [8]. However, for complex geometric structures such as grounding grid down conductors, the reflection, transmission, and mode conversion of Lamb waves are more pronounced, requiring a balance between excitation stability and robustness of signal interpretation [9,10]. Traditional piezoelectric or electromagnetic acoustic excitation methods often suffer from rapid signal attenuation and high noise levels in bent structures, prompting researchers to turn their attention to transduction methods based on the magnetostrictive effect. Magnetostrictive materials undergo length changes under the influence of a magnetic field, enabling efficient conversion between electromagnetic and mechanical energy [11,12,13,14]. In particular, rare-earth-based giant magnetostrictive materials, such as Terfenol-D and Galfenol, possess high magnetostriction coefficients and controllable stress response characteristics, exhibiting significant advantages in non-contact guided wave excitation of complex metal structures [15]. Compared to traditional materials, Galfenol offers superior mechanical properties, energy conversion efficiency, and temperature stability [16], providing a feasible pathway for establishing high-energy-density, low-loss magnetostrictive seismic sources. Although existing studies have confirmed that magnetostrictive excitation can be used for Lamb wave detection, the quantitative theory for the complex wavefield distribution in grounding grid down conductors remains insufficiently developed. The lack of a unified coupling model and signal identification mechanism has become the main bottleneck restricting the transition of current Lamb wave detection theory to practical engineering applications.

The magnetostrictive source proposed in this study adopts a loading mechanism analogous to mechanical impact. This fundamental difference in the transduction principle has two key advantages for on-site grounding grid inspection. (1) Robustness and surface adaptability: as a self-contained impactor, the magnetostrictive source is largely insensitive to common surface conditions of down conductors, such as roughness, corrosion, or concrete covering. In contrast, the coupling efficiency of piezoelectric transducers degrades rapidly with surface condition and aging of the couplant. (2) Simplified deployment: acting as a localized in-plane impactor, the source does not require precise angular alignment as needed for oblique incidence methods. Compared with conventional Lorentz-force-based EMAT transducers, this method leverages the giant magnetostrictive effect of the Galfenol alloy to achieve higher force density under a compact biased permanent magnet configuration, rather than relying solely on eddy currents in low-conductivity steel plates. Consequently, it improves the signal-to-noise ratio with a lightweight, low-power-consumption setup.

## 2. Methods

### 2.1. Basic Theory of Lamb Wave Detection

The laptop used in this study is an HP ProBook 440 G7 (HP Inc., Beijing, China). The magnetostrictive Lamb wave measurement instrument was provided by Chongqing Triloop Prospecting Technology Co., Ltd. (Chongqing, China) The magnetostrictive excitation source was developed by the Electromagnetic Detection Research Group, School of Electrical Engineering, Chongqing University (Chongqing, China). The oscilloscope model is a Tektronix MSO2024 (Tektronix, Inc., Beaverton, OR, USA).

**Figure 1:**
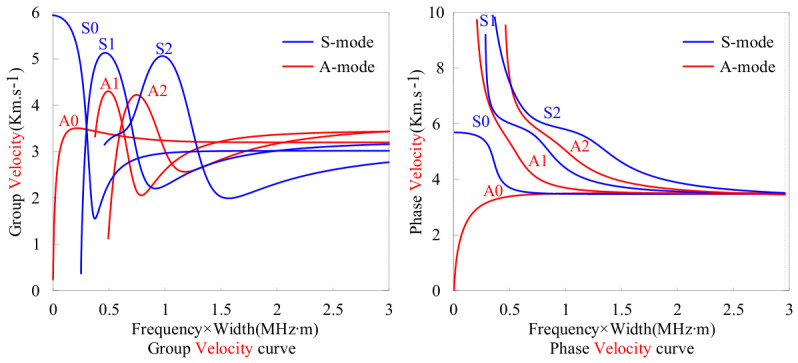
Lamb wave dispersion curves.

Lamb waves, combined with boundary conditions, lead to the Rayleigh–Lamb dispersion equations, which form the theoretical foundation for describing the modal characteristics and propagation behavior of Lamb waves. The Rayleigh–Lamb equations have no analytical solution and require numerical methods such as the Newton–Raphson method or Brent’s method to obtain the dispersion relations. The calculated results are often presented as dispersion curves, with the frequency–thickness product (*f* × *d*), where *d* is the full thickness on the horizontal axis and group velocity or phase velocity on the vertical axis, as shown in Figure 1. The physical parameters of the down conductor of the grounding grid are shown in Table 1.

Figure 1 shows the curves for 6 modes: S_0_, S_1_, S_2_, and A_0_, A_1_, A_2_. The horizontal axis is the product of frequency. When the frequency is low (below 20 kHz), only the A_0_ and S_0_ modes exist. Their phase velocities are approximately 1000~3000 m/s for A_0_ and 3000~5800 m/s for S_0_.

According to Equations (1) and (2):

(1) $$k=\frac{2\pi f}{c_{p}}$$

(2) $$\lambda =\frac{c_{p}}{f}$$

where *f* is the frequency and *λ* is the wavelength.

The A_0_ mode has a shorter wavelength, making it more prone to strong scattering from defects of comparable size; the S_0_ mode has a longer wavelength, easily bypassing defects and producing a weak response. Therefore, for defects on the plate surface or in the thickness direction, the A_0_ mode exhibits higher sensitivity and response amplitude, making it the optimal choice for low-frequency guided wave detection. Hence, this study selects the A_0_ mode as the primary mode for defect detection.

**Figure 2:**
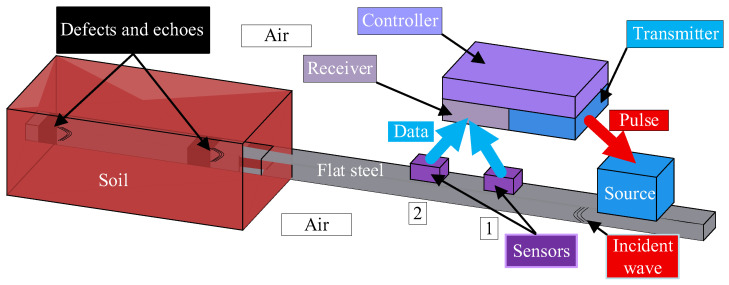
Schematic diagram of grounding grid down-conductor inspection.

Lamb wave detection is mostly carried out using an array-type arrangement of sensors. This method offers advantages such as scanning capability and improved signal resolution. Currently, most researchers and industrial products rely on array-type sensors. The excitation source is placed at the top of the down conductor, with the sensors arranged in sequence. When the excitation source is triggered, the sensors begin data acquisition. A schematic diagram of the detection setup is shown in Figure 2.

For the design of excitation sources in Lamb wave detection for grounding down conductors, the core idea is to suppress wavefield interference caused by the environment and the object under test, while enhancing the sensitivity and signal-to-noise ratio of defect signals. In this application, magnetostrictive transducers demonstrate significant advantages: their non-contact or flexible patch design adapts well to the flat steel surface of the down conductor; they readily enable low-frequency, high-energy excitation, improving penetration and anti-interference capability; and they offer strong environmental robustness, making them suitable for establishing baseline signals required for long-term monitoring. These characteristics provide a more stable and controllable wavefield input condition for separating defect signals from complex backgrounds.

### 2.2. Research on the Theory of Magnetostrictive Lamb Wave Detection

There are various magnetostrictive materials in nature, which are currently divided into two main categories: traditional magnetostrictive materials (such as nickel-based materials, iron-based alloys, and ferrites) have weak magnetostrictive properties and limited applications; new rare-earth giant magnetostrictive materials (such as Terfenol-D and Galfenol) have magnetostrictive coefficients one to two orders of magnitude higher than traditional materials. Magnetostrictive materials undergo length changes under the action of a magnetic field, converting electromagnetic energy into mechanical energy, making them suitable for ultrasonic transducers and excitation sources. Among them, Galfenol exhibits slightly lower magnetostrictive performance than Terfenol-D but better mechanical properties. The comparison between the two in this study is shown in Table 2.

Based on the performance comparison between Terfenol-D and Galfenol, Galfenol has a tensile strength (500~700 MPa) far exceeding that of Terfenol-D (28~50 MPa), offering higher mechanical reliability and impact resistance, making it suitable for long-term dynamic operation under high-frequency and high-load conditions. Although its magnetostriction coefficient (200~400 ppm) is lower than that of Terfenol-D (1000~2000 ppm), its higher relative permeability (100~200) and low coercivity (0.5~2 kA/m) significantly reduce driving energy consumption and improve response efficiency, making it particularly suitable for real-time control scenarios. Moreover, Galfenol’s wide temperature range adaptability (maximum operating temperature 350~400 °C) overcomes the high-temperature performance degradation issue of Terfenol-D, making it a better choice in terms of energy conversion efficiency, environmental adaptability, and overall engineering feasibility. Therefore, this study ultimately selects Galfenol as the magnetostrictive source material.

#### 2.2.1. Mathematical Model of Magnetostriction

The magnetostriction component along an arbitrary direction can be calculated using the nonlinear magnetization equation, the specific form of which is shown in Equation (3):

(3) $$\lambda_{i}=\frac{3}{2}\lambda_{s}(\alpha_{i}^{2}-\frac{1}{3})$$

In this equation, *λ_i_* represents the magnetostrictive elongation along the *i* direction, and its magnitude is determined by the magnetostriction constant *λ_s_* and the direction cosine of the magnetization *α_i_*. The direction cosine of magnetization *α_i_* is defined as the ratio of the magnetization intensity *M_i_* along the magnetization direction to the saturation magnetization *M_s_* of the material:

(4) $$\partial_{i}=\frac{M_{i}}{M_{s}}$$

Substituting Equation (4) into Equation (3) yields Equation (5):

(5) $$\lambda_{i}=\frac{3}{2}\lambda_{s}\left[{\left(\frac{M_{i}}{M_{s}}\right)}^{2}-\frac{1}{3}\right]$$

The term −1/3 in the above equation reflects the physical state in which the internal magnetic moments of the material are randomly oriented in the absence of an external magnetic field or prestress. In practical designs, magnetostrictive materials are usually subjected to sufficient prestress such that, at the initial stage of the magnetization process, all magnetic moments are perpendicular to the magnetization direction. Under this condition, the influence of the −1/3 term can be neglected, thereby obtaining the magnetization equation applicable to practical modeling, Equation (6):

(6) $$\lambda_{i}=\frac{3}{2}\lambda_{s}{\left(\frac{M_{i}}{M_{s}}\right)}^{2}$$

The magnetostrictive elongation in a given direction depends on the magnetostriction constant *λ_s_* and the magnetization intensity *M_i_* in that direction. For the hysteresis phenomenon, the Jiles–Atherton model can be used for description:

(7) $$M_{an}=M_{s}\left[\frac{e^{\frac{H_{e}}{a}}+e^{-\frac{H_{e}}{a}}}{e^{\frac{H_{e}}{a}}-e^{-\frac{H_{e}}{a}}}-\frac{a}{H_{e}}\right]$$

(8) $$\frac{\partial M_{irr}}{\partial \sigma }=\frac{\sigma }{E_{\xi }}(M_{an}-M_{irr})$$

(9) $$M_{rev}=c(M_{an}-M_{irr})$$

(10) $$M_{i}=M_{irr}+M_{rev}$$

(11) $$H_{e}=H_{0}+\tilde{\alpha }M_{i}$$

In the above equations, *M_an_* is the anhysteretic magnetization; *M_s_* is the saturation magnetization of the magnetostrictive material rod; *H_e_* is the effective magnetic field intensity; α is the shape coefficient of the anhysteretic magnetization of the material rod; *α* is the irreversible magnetization; σ is the axial stress applied to the material rod; E is the Young’s modulus of the magnetostrictive material; ξ is the energy coupling coefficient per unit volume; *M_rev_* is the reversible magnetization; *c* is the reversibility coefficient; in the calculation, $\tilde{\alpha }$ is usually regarded as a constant; *H*_0_ is the initial magnetic field intensity.

The relationship between magnetization intensity *M_i_* and stress *σ* is usually described using a magneto-mechanical coupling model, as shown in Equation (12):

(12) $$\frac{\partial M_{i}}{\partial \sigma }=\frac{\sigma (1-c)}{E_{\xi }}(M_{an}-M_{irr})+c\frac{\partial M_{an}}{\partial \sigma }$$

As for the relationship between magnetization intensity *M_i_* and magnetic field intensity *H*, it can be described by means of the differential magnetic susceptibility, as shown in Equation (13):

(13) $$\frac{\partial M_{i}}{\partial H}=\frac{\left(1-c\right)}{\delta k(M_{an}-M_{irr})-\tilde{\alpha }}+c\frac{\partial M_{an}}{\partial H}$$

In the above equation, *δ* = 1 is taken when the magnetic field intensity increases, and *δ* = −1 is taken when the magnetic field intensity decreases.

#### 2.2.2. Frequency Doubling Effect

The so-called frequency doubling effect refers to the phenomenon whereby the vibration frequency of a magnetostrictive rod is twice the frequency of the alternating magnetic field it is subjected to, hence the name. In magnetostrictive materials, deformation has an important characteristic: the magnetostriction coefficient λ of the material is approximately proportional to the square of the magnetic flux density **B**(t), as shown in Equation (14):

(14) $$\lambda =k(B(t))\times B{\left(t\right)}^{2}$$

where *k*(**B**(*t*)) is a proportionality function, which is a function of the magnetic flux density **B**(*t*).

Magnetostrictive materials deform due to the internally generated magnetostrictive stress *T_m_*. According to Hooke’s law, Equation (15) can be obtained:

(15) $$T_{m}=E\times \lambda$$

where *E* is the Young’s modulus of the magnetostrictive material. Substituting Equation (14) into Equation (15) yields Equation (16):

(16) $$T_{m}=E\times k(B(t))\times B{\left(t\right)}^{2}$$

It can thus be seen that the magnitude of the magnetostrictive stress Tm has a quadratic functional relationship with the magnetic flux density **B**(*t*). We assume that the driving magnetic field is a magnetic field that varies periodically with time, as shown in Equation (17):

(17) $$B(t)=B_{m}(t)\sin(\omega t)$$

Substituting Equation (17) into Equation (16) yields Equation (18):

(18) $$T_{m}=F(B(t))\times B_{m}(t)\times \sin(\omega t)=F(B(t))\times B_{m}(t)\times \frac{1-\cos(2\omega t)}{2}$$

In the above equation, treating the two terms *F*(**B**(*t*)) × **B***_m_*(*t*) as a constant *k*′ allows it to be written as Equation (19):

(19) $$T_{m}=\frac{K^{\prime }}{2}-\frac{K^{\prime }}{2}\cos(2\omega t)=T_{0}+T_{2\omega }$$

**Figure 3:**
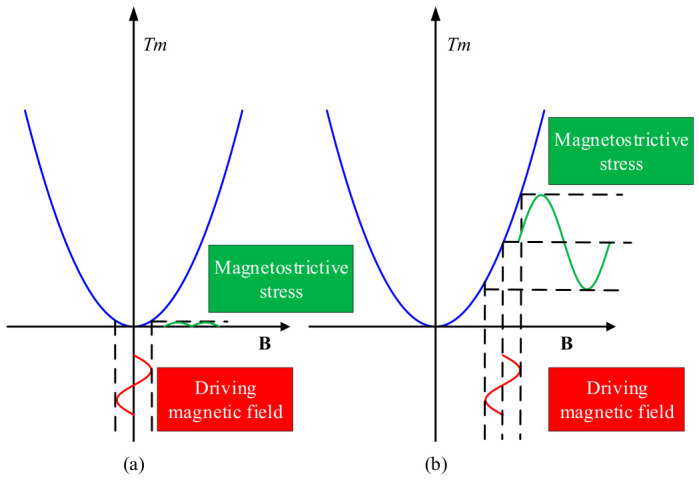
Elimination of the frequency doubling effect by a bias magnetic field. (**a**) Without bias magnetic field; (**b**) With bias magnetic field.

It can be seen that, in a magnetostrictive rod, the magnetostrictive stress *T_m_* generated by the driving magnetic field contains two parts: one part is the constant stress *T*_0_, and the other part is the alternating stress *T*_2*ω*_, and the frequency of the alternating stress is exactly twice the frequency of the driving magnetic field; hence, it is called the frequency doubling effect. In the Lamb wave corrosion detection of grounding grid down-leads, the magnetostrictive vibration source is required to achieve a single precise vibration, and the frequency doubling effect can seriously interfere with the experimental detection results, so methods need to be adopted to eliminate it. The frequency doubling effect can be eliminated by applying an external bias magnetic field, that is, by pre-applying a constant magnetic field to the magnetostrictive material rod. This can both achieve the goal of eliminating the frequency doubling effect and enable the magnetostrictive vibration to achieve same-frequency vibration. The generation principle of the frequency doubling effect and the principle of eliminating the frequency doubling effect using a bias magnetic field are shown in Figure 3.

**Figure 4:**
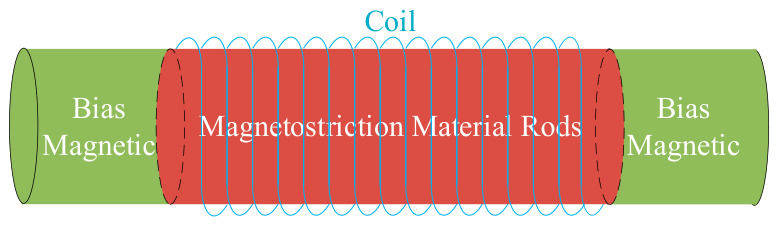
Structure diagram of the magnetostrictive source.

There are two main ways to provide a bias magnetic field for magnetostrictive materials: one is to use permanent magnets, and the other is to use a DC energized coil. In comparison, the bias magnetic field provided by permanent magnets has higher stability, and offers advantages such as small size, compact structure, and no need for an additional DC power supply. Therefore, this study selects permanent magnets as the bias magnetic field source for the vibration source. A bias magnetic cylinder can also provide a bias magnetic field, but it will significantly increase the overall volume of the vibration source, severely affecting the portability of the equipment. Based on the above considerations, the bias magnetic field design scheme adopted in this study is shown in Figure 4. The vibration-generating structure consists of three core parts: a magnetostrictive material rod in the middle, a current excitation coil sleeved over the rod, and bias magnets placed at both ends of the rod. A bipolar pulse current is injected into the coil, generating an alternating magnetic field. Under the action of the bias magnetic field, at the moment of turn-off, it rapidly strikes the down-lead flat steel.

In terms of excitation coil selection, the solenoid coil has significant advantages over the Helmholtz coil. The reasons are as follows: first, the solenoid can generate a higher magnetic flux density in the local space tightly wrapped around the magnetostrictive rod, with a concentrated magnetic circuit and low magnetic flux leakage. This leads to a higher degree of magnetization of the material, thereby achieving greater magnetostrictive strain and higher Lamb wave amplitude. Under the same number of turns, current, and power conditions, the local peak magnetic field generated by the solenoid coil is generally significantly higher than that of a Helmholtz coil designed for a uniform field. Second, the excitation region of the solenoid is compact, enabling efficient mechanical coupling between the magnetostrictive rod and the test plate. This makes the excitation approximate a controlled line source or area source, facilitating the optimization of Lamb wave mode selection and beam directivity through the length, cross-section, and arrangement of the rod. In contrast, the advantage of the Helmholtz coil lies in generating a large-volume uniform magnetic field, which is more suitable for the uniform excitation of multiple or distributed devices, but it is inefficient for locally exciting a single magnetostrictive rod. Third, from an electrical characteristic perspective, the solenoid has a compact structure, relatively low inductance, and small parasitic parameters, which is conducive to exciting Lamb waves while maintaining a stable waveform and bandwidth. The Helmholtz coil, however, has larger dimensions, higher inductance and losses, often requiring higher driving voltage and power in the same frequency range, and it is prone to self-resonance and reduced efficiency. Considering factors such as magnetic field strength, energy coupling efficiency, frequency band characteristics, and structural compactness, for nondestructive testing systems that use a single or only a few magnetostrictive elements to excite Lamb waves, the solenoid coil is generally superior to the Helmholtz coil in excitation efficiency and engineering feasibility.

The Neodymium–Iron–Boron (NdFeB) permanent magnet material is selected for the bias magnets. NdFeB magnets are currently the strongest permanent magnets available. Using them to provide the bias magnetic field can eliminate the complex bias magnetic field debugging steps required in the practical application of magnetostrictive vibration sources, thus making their use more convenient.

## 3. Experimental Verification of Magnetostrictive Lamb Wave Detection

Based on the above conclusions, this study only needs to consider signals in the range of 0~20 kHz, so a sensor with a bandwidth of 20 kHz was selected. The instrument has a sampling rate of 1.25 Msps, and the number of sampling points per channel is 8192.

**Figure 5:**
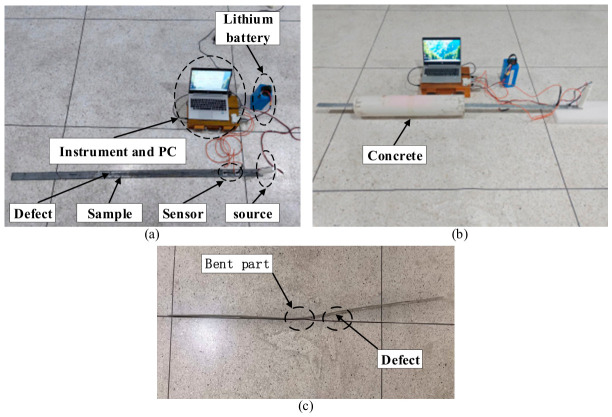
Experimental verification of the magnetostrictive source: (**a**) Sample 1; (**b**) Sample 2; (**c**) Sample 3.

Three types of grounding grid down-lead flat steel samples were selected to test the magnetostrictive vibration source, as shown in Figure 5.

The sample parameters are shown in Table 3.

The parameters of the magnetostrictive vibration source and excitation are shown in Table 4.

**Figure 6:**
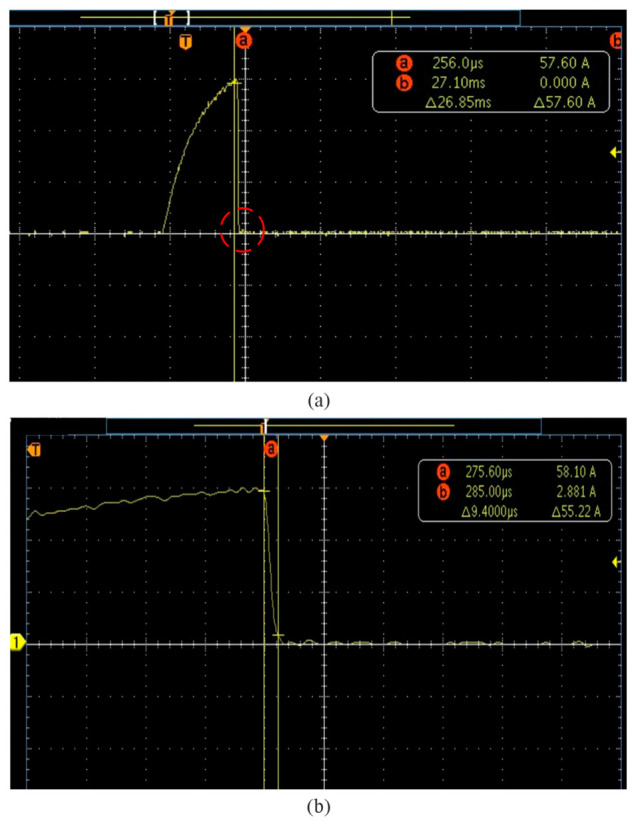
Magnetostrictive source excitation: (**a**) Transmitted current waveform; (**b**) Close-up of turn-off portion.

The magnetostrictive vibration source can only achieve an instantaneous force under step excitation. However, its wide frequency band will simultaneously excite signal components of multiple frequencies. After superposition, highly complex signals will be generated, making defect echoes difficult to identify. The transmitting current and signal spectrum are shown in Figure 6.

**Figure 7:**
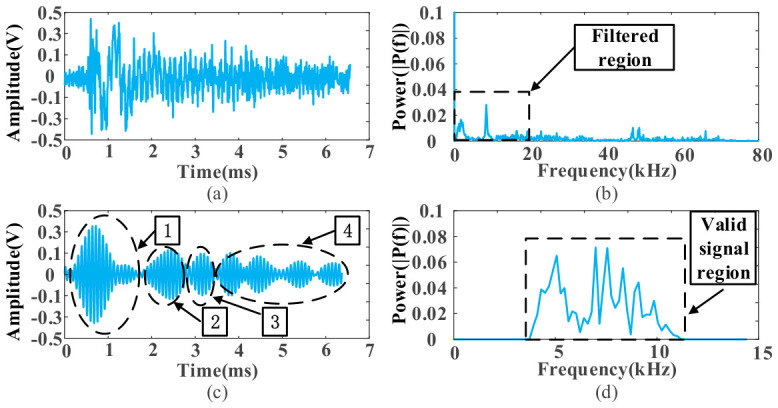
Detection signal of Sample 1: (**a**) Original signal; (**b**) Spectrum of the original signal; (**c**) Filtered signal; (**d**) Spectrum of the filtered signal.

**Figure 8:**
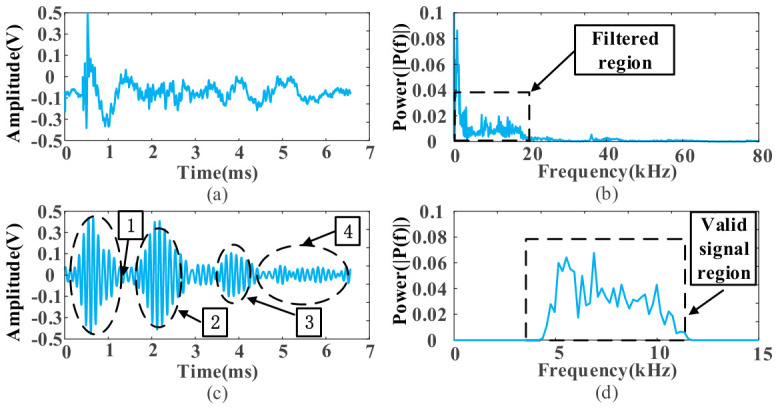
Detection signal of Sample 2: (**a**) Original signal; (**b**) Spectrum of the original signal; (**c**) Filtered signal; (**d**) Spectrum of the filtered signal.

**Figure 9:**
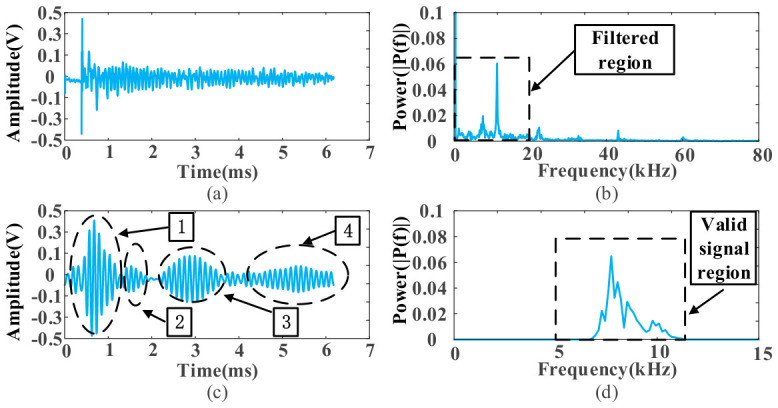
Detection signal of Sample 3: (**a**) Original signal; (**b**) Spectrum of the original signal; (**c**) Filtered signal; (**d**) Spectrum of the filtered signal.

Based on Figure 6a, which is the transmitting current waveform, and Figure 6b, which is a close-up of the turn-off portion within the red circle in Figure 6a, the turn-off time is approximately 9.4 μs. Under this excitation signal, the excited Lamb wave reception signals are shown in Figure 7, Figure 8 and Figure 9.

## 4. Discussion

From Figure 7a, it can be seen that there are obvious response and decaying oscillation components in the original signal, indicating that the Lamb waves excited by the magnetostrictive source can effectively propagate in the flat steel and be collected by the receiving end. However, the original signal is mixed with considerable noise and spurious frequency components, causing the characteristics of each echo to be insufficiently clear. Figure 7b shows that the original signal energy is mainly distributed in the low-frequency range. Considering that the sensor bandwidth is 20 kHz, the 10~20 kHz band was selected as the band-pass filter band to retain the main Lamb wave information and suppress irrelevant interference. After filtering, the echo structure in Figure 7c is enhanced and can be relatively clearly divided into four regions. Region 1 arrives earliest, with the largest amplitude, and it can be identified as the direct wave, indicating that the magnetostrictive source can achieve effective excitation in the flat steel and the propagation signal can be received. Region 2 is located after the direct wave. Combined with the known condition that the defect in Sample 1 is located 1200 mm from the end, it can be preliminarily identified as a defect echo, indicating that the Lamb wave can reflect off the defect and form an echo signal during propagation. Region 3 appears later. In conjunction with the structural characteristic that the total length of the specimen is 2000 mm, it can be considered as the end reflection echo formed after the Lamb wave propagates to the far end. Region 4 is mainly distributed in the later part of the signal, and its waveform is relatively dispersed. It is speculated to be a composite echo formed by multiple reflections, mode conversions, and spurious superpositions of Lamb waves at the defect, ends, and boundaries. Figure 7d shows that the frequency spectrum distribution of the filtered signal is more concentrated, effectively suppressing high-frequency noise and irrelevant clutter, and improving the clarity of echo identification.

From Figure 8a, it can be seen that the Lamb waves excited by the magnetostrictive source can effectively propagate in concrete-covered flat steel. The obvious wave packet superposition phenomenon in the original signal indicates that the concrete covering and interface mutation have an impact on Lamb wave propagation. Figure 8b shows that the signal energy is mainly concentrated in the low-frequency range. Considering the sensor bandwidth of 20 kHz, the 10~20 kHz band was selected as the band-pass filter band to retain effective Lamb wave information and suppress irrelevant noise. After filtering, four relatively distinct echo regions can be observed in Figure 8c. Region 1 arrives earliest with the largest amplitude, and can be identified as the direct wave. Regions 2 and 3 are located in the middle time period. Combined with the structural characteristics that the concrete-to-air interface in the sample is located at 1000 mm and the defect is at 1200 mm, they can be considered as mainly corresponding to the composite response of the interface reflection echo and the defect reflection echo. Because the spatial positions of the two are close, and under concrete-covered conditions, Lamb wave propagation is more prone to wave packet broadening and multipath superposition; the interface echo and defect echo may overlap in the time domain and be difficult to completely separate. This is also an important reason why only four distinct echo regions appear in the filtered signal. Region 4 appears at a later time. Combined with the boundary condition that the total length of the specimen is 2000 mm, it can be considered as the far-end end echo and its subsequent multiple reflection clutter. From Figure 8d, it can be seen that the frequency spectrum distribution of the filtered signal is more concentrated, spurious frequency components are effectively suppressed, and the clarity of echo identification is significantly improved.

From Figure 9a, it can be seen that the bending region has a significant impact on Lamb wave propagation. From Figure 9b, it is known that the original signal energy is mainly concentrated in the low-frequency range. Considering the sensor bandwidth of 20 kHz, the 10~20 kHz band was selected as the band-pass filter band to retain the main Lamb wave information and suppress irrelevant interference.

After filtering, four distinct echo regions can be relatively clearly identified in Figure 9c. Region 1 arrives earliest with the largest amplitude, and can be identified as the direct wave. Region 2 is located after the direct wave. Combined with the structural characteristic that Sample 3 has a bending region 950 mm from the end, it can be identified as an echo from the bend, indicating that Lamb waves have good sensitivity to geometric mutations. Region 3 appears after Region 2. Combined with the known condition that the defect is located 1200 mm from the end, it can be considered a defect echo, indicating that the Lamb wave can still interact with local defects and form identifiable reflection signals after passing through the bending region. Region 4 is located later. Combined with the boundary condition that the total length of the specimen is 2000 mm, it can be considered as the far-end end echo and other multiple reflection clutter.

From Figure 9d, it can be seen that the frequency spectrum distribution of the filtered signal is more concentrated, spurious frequency components are effectively suppressed, and the clarity of echo identification is significantly improved.

Synthesizing Figure 7, Figure 8 and Figure 9, it can be seen that the magnetostrictive Lamb wave detection system constructed in this study can achieve stable Lamb wave excitation and effective reception in long straight flat steel specimens, and obtain response signals with clear physical meaning such as direct waves, defect echoes, and end echoes, verifying the feasibility of this method for Lamb wave detection in flat steel components.

## 5. Conclusions

This study proposed a novel Lamb wave excitation source technology based on the magnetostrictive mechanism and systematically studied it in terms of theoretical modeling, material selection, excitation mode, modal analysis, and multiphysics simulation.

First, considering that grounding grid down-lead flat steel has the characteristics of a typical plate-like waveguide structure, the Rayleigh–Lamb dispersion theory was introduced to analyze the propagation law of Lamb waves in flat steel, clarifying the applicability of low-order modes in the detection of such structures. By comparing the propagation characteristics of the A_0_ and S_0_ modes, it was found that the A_0_ mode is more conducive to improving the defect response capability under the same frequency conditions, so it was adopted as the primary mode. On this basis, this study constructed an “electro-magneto–mechanical–wave” coupling model for magnetostrictive Lamb wave excitation, elucidating the energy conversion process: the step current pulse in the coil generates a transient magnetic field, the magnetic field acts on the magnetostrictive material causing rapid strain and stress output, and, through mechanical coupling, acts on the surface of the flat steel, ultimately exciting Lamb waves. This model reveals the intrinsic relationship among excitation current, magnetic field intensity, magnetostrictive strain, output load, and Lamb wave amplitude. It also points out that the relationship between magnetostrictive strain and magnetic field intensity exhibits significant nonlinearity due to the magnetization process, hysteresis, and saturation effects, thus requiring reasonable current parameters, magnetic circuit structure, and material selection to improve energy conversion efficiency and excitation stability.

In terms of materials, this study compared the performance characteristics of typical magnetostrictive materials and finally selected the Iron–Nickel alloy Galfenol as the core material. It possesses good magnetostrictive performance, mechanical strength, toughness, impact resistance, and environmental adaptability, making it more suitable for the requirements of repeated installation, loading, and complex environments in the on-site detection of grounding grid down-leads.

Regarding the excitation method, a step pulse current-driven coil can establish a transient magnetic field in a short time. Its application to flat steel samples enables the relatively stable excitation of Lamb wave signals, confirming the validity of both the theory and the structural design.

## Figures and Tables

**Table 1:** Grounding grid down conductor parameter settings.

*h* (mm)	*ρ* (kg/m^3^)	*E* (Pa)	*υ*	*c_L_* (m/s)	*c_T_* (m/s)
2	7.85 × 10^3^	2 × 10^11^	0.3	5900	3200

*h* is half-thickness, *ρ* is density, *E* is Young’s modulus, *ν* is Poisson’s ratio, *c_L_* is longitudinal wave velocity, and *c_T_* is transverse wave velocity.

**Table 2:** Key performance parameter indicators of Terfenol-D and Galfenol.

	Material	Terfenol-D	Galfenol
Performance Indicator			
Magnetostriction coefficient *λ* (ppm)		1000~2000	200~400
Relative permeability *μ*r		10~50	100~200
Tensile strength Rm MPa)		28~50	500~700
Remanent induction Br (T)		0.8~1.0	0.5~1.2
Coercivity bHc (kA/m)		1~5	0.5~2
Intrinsic coercivity iHc (kA/m)		5~10	2~5
Maximum energy product BH (kJ/m^3^)		20~50	10~30
Maximum operating temperature Tw (°C)		150~200	350~400

**Table 3:** Parameters of test samples.

	Length (mm)	Width (mm)	Thickness (mm)	Defect Position (mm)	Concrete Interface (mm)	Bending Position (mm)
Sample 1	2000	60	4	1200	0	0
Sample 2	2000	60	4	1200	1000	0
Sample 3	2000	60	4	1200	0	950

**Table 4:** Parameters of the magnetostrictive source and excitation.

Length (mm)	Diameter (mm)	Coil Turns (N)	Transmitting Current (A)	Transmitting Frequency (kHz)	Turn-off Time (μs)
750	25	30	±30	1	9.4

## Data Availability

The data presented in this study are available on request from the corresponding author because they involve core data and are not convenient to upload. If you have any requests, please contact the corresponding author. We appreciate your understanding.
